# Subcutaneous administration of the malaria R21/Matrix M vaccine and immune complex formation with anti-circumsporozoite protein mAb 2A10 elicit protective efficacy in mice

**DOI:** 10.3389/fimmu.2025.1675780

**Published:** 2025-10-07

**Authors:** Ekta Mukhopadhyay, César López Camacho, Adrian V. S. Hill, Ahmed M. Salman

**Affiliations:** The Jenner Institute, University of Oxford, Oxford, United Kingdom

**Keywords:** vaccine, malaria, immune-complex, antibody, protection

## Abstract

**Introduction:**

R21, the most efficacious malaria vaccine to date, has been recommended by the World Health Organization (WHO) for the prevention of malaria in children. The current vaccination schedule requires three intramuscular doses per year. Optimizing vaccine administration strategies, including exploring alternative routes of immunization and novel vaccine formulations, has the potential to reduce the number of required doses to achieve high efficacy. Immune complexes (ICs), formed by combining antigens with their cognate antibodies, have been successfully employed in licensed poultry vaccines for viral diseases and are showing promise in preclinical studies for human viral vaccines. Co-delivery of antigen with immune complexes has been reported to enhance antibody titers in preclinical models.

**Methods:**

Here, we present the first report of the immunogenicity and short- term high protective efficacy of R21/Matrix-M administered via the subcutaneous (SC) route, as well as in a modified formulation as an immune complex (IC) (R21: anti-NANP mAb 2A10) with only two immunizations. We also evaluated co-administration of R21 with pre-formed ICs.

**Results:**

R21/MM administered via the SC route is immunogenic and more efficacious (100% in BALB/c mice) than the IM route. R21:2A10 IC/MM is immunogenic and induces sterile protection in BALB/c mice. Co-administration of R21/MM with R21:2A10 IC is immunogenic but less protective than IC/MM alone in BALB/c mice.

**Conclusion:**

While IC-based vaccination strategies have primarily been explored for viral diseases, this study represents the first application of this approach to a parasitic disease. Our findings provide new insights into the potential of alternative vaccine delivery strategies and immune complex platforms for improving malaria vaccination outcomes.

## 1 Introduction

Malaria continues to pose a significant global health burden, with over 240 million cases and 608,000 deaths reported across 85 countries in 2022 (1). The rollout of the RTS,S/AS01 vaccine and the recent World Health Organization’s (WHO) recommendation of the R21/Matrix-M (R21/MM) vaccine, both for use in children, represent significant milestones in malaria control efforts. However, the dosing regimen for both vaccines require three or more doses, so that alternative administration strategies that can reduce the number of doses required might be desirable. Malaria elimination remains a key goal of global health initiatives, with strategies focused on increasing vaccine coverage, minimizing waste, and maximizing the efficiency of available resources. Reducing the number of required vaccine doses could also significantly impact these efforts by decreasing vaccine wastage and improving the logistics of distribution, especially in remote or resource-constrained areas. With fewer doses needed per individual, more people could receive complete vaccination schedules, thereby increasing overall population coverage. This could be particularly impactful in regions with high malaria burden and limited healthcare infrastructure, where logistical challenges often hinder the full utilization of vaccines. Simplified vaccine schedules could also enhance adherence, reduce associated costs, and accelerate the path toward malaria elimination.

Subcutaneous route of vaccine administration has been explored for malaria with promising outcomes. Subcutaneous immunizations of BALB/c mice with axenic Plasmodium yoelii, conferred sterile protection against P. yoelii infectious sporozoite challenge (2). In a pre-clinical study in C57/BL6 mice, full length *Plasmodium falciparum* Circumsporozoite protein administered via SC route with adjuvant Long chain poly (I.C) show 50% protection on challenge (3). Immune complex (IC) vaccination, which involves the administration of a pre-formed antigen-antibody complex, is a strategy with potential to enhance immunogenicity and efficacy. First explored over a century ago, this approach was initially developed using antisera complexed with bacterial toxoids to reduce side effects in human vaccines (4). Naturally, ICs are formed *in vivo* when antigens bind to circulating antibodies, leading to pathogen elimination through phagocytosis or complement activation (5). ICs interact with follicular dendritic cells (FDCs) via the Fc domain of the antibody, enabling antigen processing and presentation via MHC molecules to T cells. This interaction facilitates B cell activation and antibody production (6).

Complement C3 complexes also assemble with ICs via complement receptors CR1 and CR2, playing a critical role in generating B cell memory (7). FDCs uniquely trap ICs, retaining them for extended periods and preventing degradation. This prolonged antigen availability supports germinal center (GC) reactions and enhances B cell memory formation through mechanisms such as the generation of iccosomes—immune-complex-coated bodies released from FDCs and subsequently internalized by B cells (8, 9).

IC-based vaccines have been successfully employed in veterinary medicine, particularly for the prevention of infectious bursal disease (e.g., Transmune IBD^®^ and Bursaplex^®^) and Newcastle Disease Virus (NDV) in poultry. Licensed in over 75 countries, these vaccines leverage freeze-dried live attenuated viruses in combination with specific antisera (10, 11). For human diseases, IC vaccination has shown promise in preclinical studies for HIV, chronic HBV, influenza, Ebola, and cancer, as well as for viral diseases like Zika and HPV, where co-delivery of virus-like particles (VLPs) with recombinant immune complexes (RICs) resulted in 2-5-fold increases in antibody titers correlating with enhanced virus neutralization (12–14). ICs have also been explored with adenovirus-based vaccines, demonstrating improved immune responses by extending antigen availability (4, 15). In human studies, HBsAg-HBIg ICs have been found safe and immunogenic, inducing potent anti-HBs responses (16).

Despite its success in viral disease models, IC-based vaccination has not been explored for parasitic diseases. R21/MM, recently licensed malaria vaccine, has been shown to induce sterile protection in preclinical models with three intramuscular (IM) immunizations (17, 18). This short -term study aims to evaluate the immunogenicity and protective efficacy of R21/MM administered via the subcutaneous (SC) route, using only two doses, and to assess whether these responses are enhanced when delivered as an IC or co-administered with R21 + IC/MM via both IM and SC routes.

## 2 Materials and methods

### 2.1 Formation of R21: mab 2A10 Immune complex

Different concentrations of anti-NANP IgG mAb 2A10, were mixed with a fixed concentration of R21 (1µg) in separate tubes to achieve the target Ab: Ag ratio (1:1, 1:2, 2:1 and 5:1), and incubated at 37°C for 1 hour, 50 rpm.

### 2.2 Confirmation of IC formation

#### 2.2.1 Western blotting

After incubation, a sample was taken into a separate tube and processed with reducing agent (Invitrogen™ NuPAGE™ Sample Reducing Agent (10X) NP0009 and Invitrogen™ NuPAGE™ LDS Sample Buffer (4X) NP0007) by incubation at 100°C for 15 minutes. Reducing agent was added to another sample from each tube, just before loading onto the gel, but not subjected to high temperature incubation to keep the buffer and running conditions the same and not denaturing the IC. The R21 control (un-complexed) was processed the same way. The samples and R21 positive control were run on NuPage 4-12% Bis- tris Midi gel (Invitrogen™ NP0336BOX) alongside pre-stained colour marker 245 kDa (NEB# P7712). Western blotting was performed using 2 membranes (1 and 2) using Transblot Turbo (Bio- Rad) on 0.2µm nitrocellulose membrane (# 1704158 and # 1704159) and the membranes were blocked in 2% BSA/PBS for 1 hour at RT on a rocking platform. Membrane 1 was incubated in primary antibody anti- HBsAg antibody (Mouse anti- Hepatitis B Surface Antigen antibody (Genetex, GTX40707)) to detect the R21 as monomer or in the complex. Membrane 2 was incubated with anti- IgG antibody linked to alkaline phosphatase (Sigma, A3562) to detect the mAb 2A10 in the IC. After incubation at RT for 1 hour on rocking platform, the membranes were washed 3 x with PBS/T and 1 x with PBS. Membrane 1 was incubated in anti- IgG antibody linked to alkaline phosphatase (Sigma, A3562) and incubated at RT for 1 hour on a rocking platform. Both the membranes were washed as above and developed using BCIP/NBT tablet (Merck, 11697471001) dissolved in 10mL of distilled water.

#### 2.2.2 Immune gold TEM

A sample of the IC from all ratios was sent to the Dunn School of Pathology Electron Microscopy Facility (University of Oxford) and processed for negative staining after tagging the IC with anti-mouse antibody conjugated to gold particles, which bound to the Fc region of the antibody forming a part of the IC. Un-complexed R21 VLP was used as a negative control to which no gold conjugated antibody would bind. 10 µl of the sample (diluted 1:10 with water) were adsorbed onto a freshly glow discharged carbon filmed 300 mesh copper grid (TAAB Laboratories, #C267) for 2 mins. Grids were then gently blotted and incubated on a 40 µl droplet of blocking buffer (0.2% porc gelatin (Sigma #G2500) in PBS) for 5 min. Grids were then blotted and incubated on 40 µl goat anti-mouse secondary antibody conjugated to 5 nm colloidal gold (Abcam, #ab27244), diluted 1:20 with blocking buffer, for 30 min. Grids were then blotted, washed 3x 2min with block buffer, then 3x 2 min with PBS before being fixed with 0.1% glutaraldehyde (Agar Scientific, #R10200) in PBS for 15 min. Grids were then washed 4x 2 min with water, then stained with 2% uranyl acetate (Agar Scientific, #R1260A) for 30 sec, blotted and air dried. All steps were performed at room temperature. Grids were imaged with a Thermo Fisher Tecnai T12 transmission electron microscope, operated at 120kV and equipped with a Gatan OneView digital camera.

### 2.3 Vaccination and sporozoite challenge

#### 2.3.1 Animals

All animal work was conducted in accordance with the UK Animals (Scientific Procedures) Act 1986 and approved by the University of Oxford Animal Care and Ethical Review Committee. Animals were group housed in individually ventilated cages under specific pathogen free conditions, with constant temperature, humidity and with a 12:12 light-dark cycle (8am to 8 pm). For induction of short-term anesthesia, animals were anaesthetized using vaporized IsoFlo^®^ (3.5%, 2Litre/minute oxygen). All animals were humanely sacrificed at the end of each experiment.

#### 2.3.2 Vaccination and blood sampling

**Figure 1:**
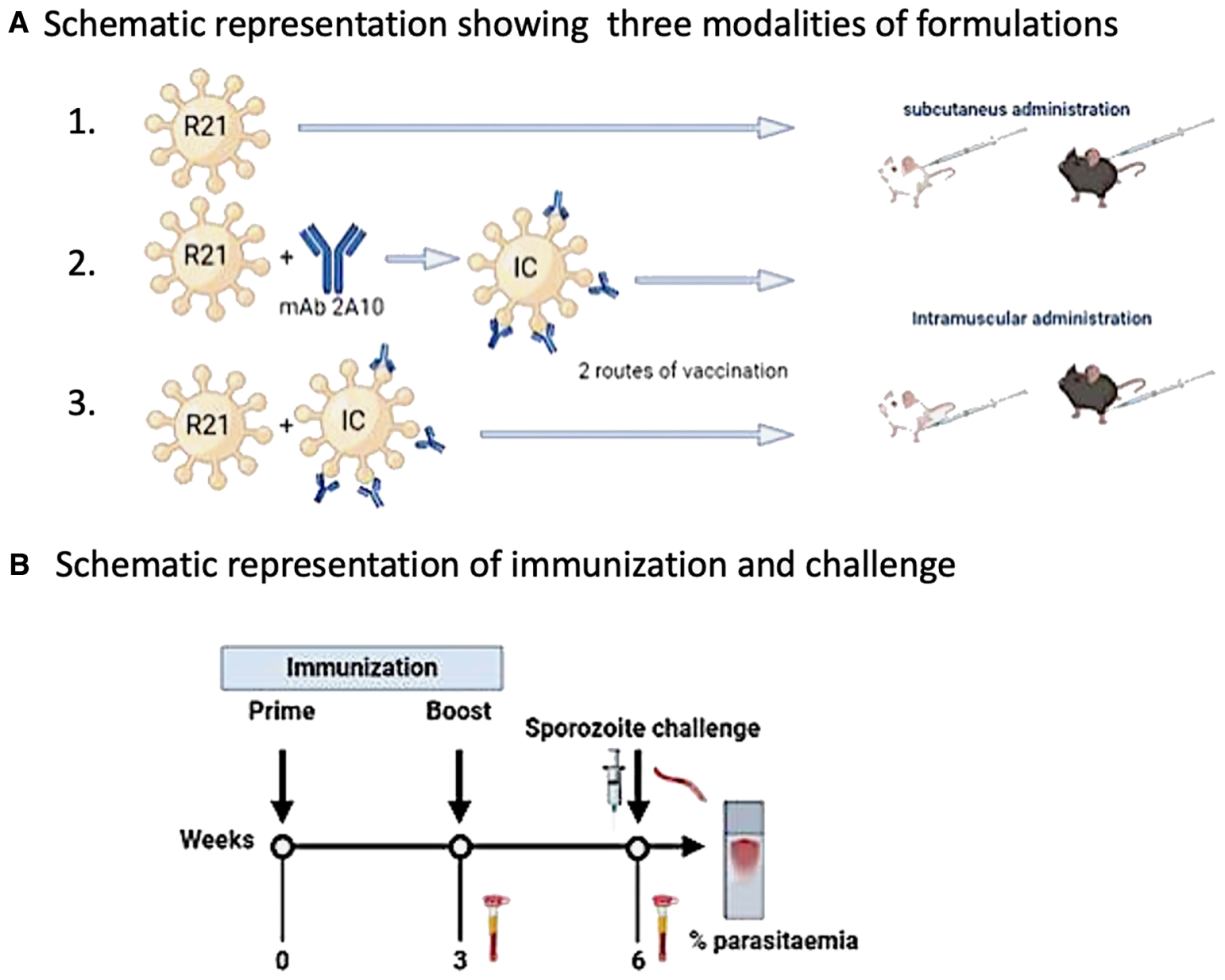
R21 vaccination regimes and assessment of efficacy against malaria challenge. Schematic representation showing three modalities of formulations **(A)** for both intramuscular and subcutaneous immunisation. (1) Un-complexed (R21), (2) Complexed with anti-NANP mAb 2A10 as an immune complex (R21:anti-NANP), and (3). Co-administered with R21: (R21 + R21:anti- NANP) BALB/c and C57/BL6 mice were subjected to an immunisation protocol of a prime and a boost at 3-week interval. Three weeks post boost, an intra-venous sporozoite challenge (1000 spz/per mouse) at three weeks post boost **(B)**. To assess the efficacy of vaccination, blood smears were sampled for determining the levels of parasitaemia. Blood stage parasitaemia was checked from day 5 post challenge and 1% parasitaemia was used as humane end point. Finally, sera was collected at 3 weeks post prime and 3 weeks post boost immunizations to analyse anti- NANP antibody titres by ELISA.

Mice were vaccinated with R21/MM three ways 1) subcutaneously, 2) as IC where 1:1 ratio of R21: 2A10 in MM, 1 µg each was used to compare with the group with 1 µg of un-complexed R21 in adjuvant and 3) co-administered where 1 µg of un-complexed R21 was mixed with IC (1:1 ratio of R21: 2A10, 1 µg each) (**Figure 1A**). 5 µg of adjuvant Matrix-M (MM) was used as recommended by Novavax Inc for all vaccinations. The volumes were made up with sterile PBS when required. Vaccination was performed within 1 hour of formulation preparation which was kept on ice till injected. Mice were vaccinated (prime) and boosted 3 weeks post prime. Blood sampling was done 3 weeks post prime and 3 weeks post boost. Blood samples were collected via tail vein bleed in a microcuvette tube (**Figure 1B**). Blood was allowed to clot by storing it at 4°C overnight before centrifuging it at 13000 rpm for 10 minutes to separate the sera. Sera were removed in individual tubes and stored at -20°C until use.

#### 2.3.3 Sporozoite production and challenge

Frozen *P. berghei* pRBC of the chimeric parasite line PbANKA-PfCSP(r)PbCSP (2257 cl2) were thawed and 100-300 µl injected intra-peritoneal (IP) into a naive BALB/c mouse. Five to six days later the parasitaemia and gametocytaemia were determined by analysing Giemsa stained thin blood films and addition of a drop of blood to exflagellation media enabled the analysis of exflagellation. Anaesthetized mice were placed onto pots of starved 4–7 days old female *Anopheles stephensi* mosquitoes for approximately 10–15 minutes. Mosquitoes infected with the chimeric *P. berghei* parasite were maintained at 19- 23°C in a humidified incubator and fed on fructose solution. At approximately 21–23 days post-feed mosquito salivary glands were dissected to obtain infectious sporozoites in the appropriate cell culture media and homogenized on ice. Sporozoite numbers were counted using a haemocytometer under phase contrast. To test the efficacy of the vaccines, vaccinated and naïve control mice groups were injected with 1000 sporozoites by intravenous (IV) injection into the lateral tail vein approximately 3 weeks post the boost vaccination (**Figure 1B**). Mice were monitored from day five post-injection via thin blood films and sacrificed when parasitemia reached 1%, calculated using linear regression. If thin blood films were negative fourteen days post-infection mice were classes as “protected” and were sacrificed.

### 2.4 Quantification of immune response using ELISA

Nunc Maxisorp plates (Invitrogen™ 44-2404-21) were coated with 100µl/well of 2µg/ml NANP peptide (6 repeats, Mimitopes, 3772801) in coating buffer (Carbonate/Bicarbonate buffer pH 9.6), sealed with clingfilm and stored at + 4°C overnight. Next day, the plates were washed 6x with PBS-Tween (0.05%). The plates were then blocked 200µl/well with 2% BSA/PBS and incubated for 1 hour at RT. Serum samples were thawed and diluted in 1% BSA-Tween. Anti NANP 2A10 mAb was used as a positive control and naïve samples were used as negative control. The blocking solution was discarded the pre- diluted serum samples in triplicates and positive control in duplicates, were added and plates were incubated for 1 to 2 hours at RT. Plates were then washed 6x with PBST and goat anti-mouse alkaline phosphatase (Sigma- Aldrich, A3562, 1 in 3000) diluted in 1% BSA-Tween was added to each well and incubated for 1 hour at RT. The plates were washed 6x with PBST and 1x with PBS. pNPP substrate (Sigma- Aldrich, 20-016) was added to each well and plates were read using Gen 5 software at 405nm.

### 2.5 Statistical analysis and software

Statistical analysis was performed using GraphPad Prism version 9 (Graphpad, USA). Mann-Whitney rank test was used for comparing two non-parametric groups. Survival and protective efficacy to in challenge experiments was presented using Kaplan-Meier curves and significance tested using the Log-Rank (Mantel-Cox) Test. The value of p< 0.0500 was considered statistically significant.

## 3 Results

### 3.1 R21 VLP can be complexed with anti-NANP mAb 2A10 in different ratios

**Figure 2:**
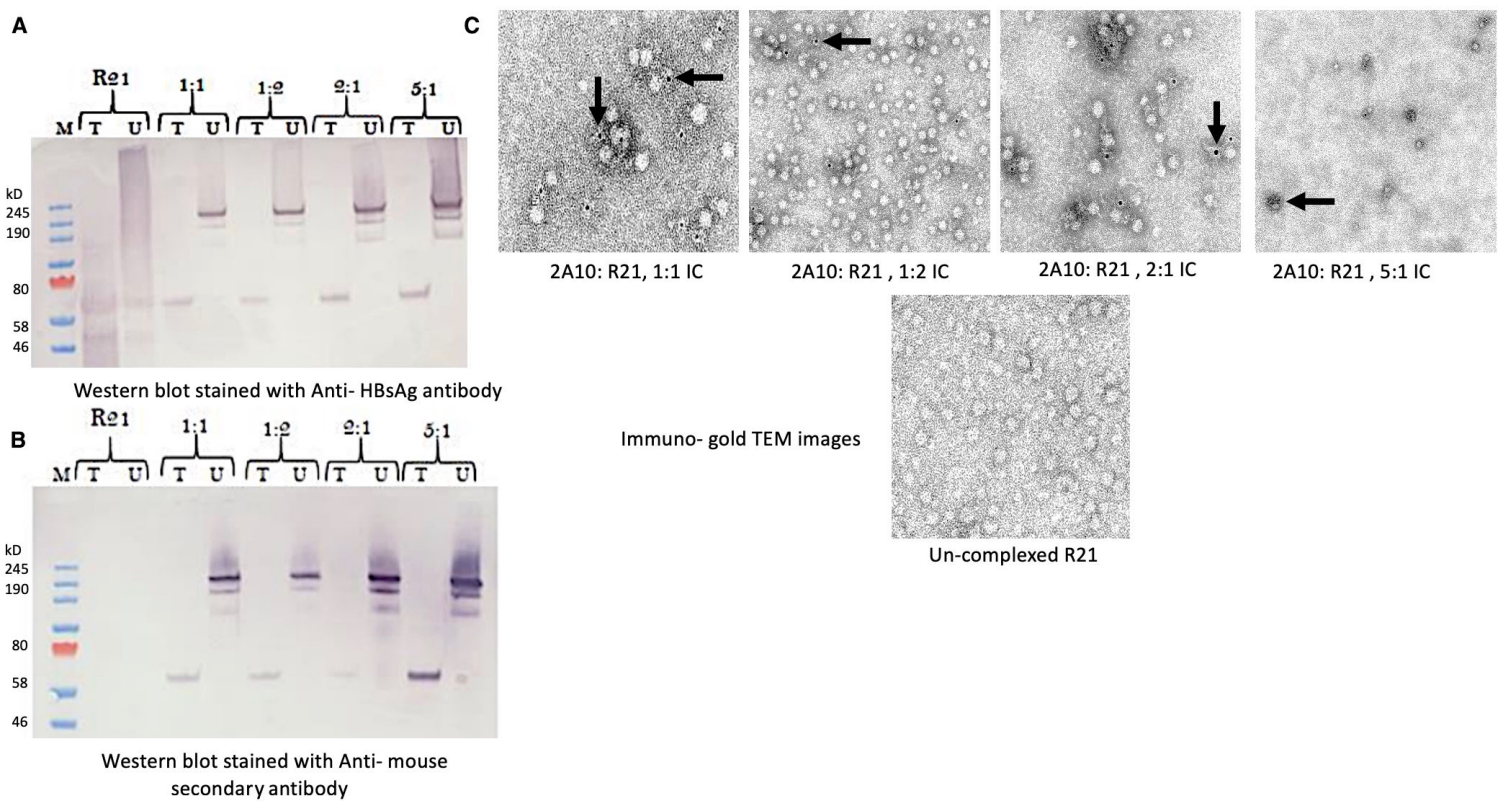
Confirmation of R21: mAb 2A10 immune complex (IC) formation: Four different ratios of anti- NANP mAb 2A10:R21 (1:1, 1:2, 2:1 and 5:1) incubated as described earlier to form an antibody: VLP immune complex. For western blot, the IC were treated (T) and un-treated (U) under reducing conditions and the blot was stained with **(A)** anti-HBsAg antibody to stain the HBsAg component of the R21 VLP and **(B)** with anti-mouse secondary antibody to stain the anti- NANP mAb 2A10 complexed to R21 forming an IC of high molecular weight (~200kD), seen in both blots **(A–C)**. **(C)** Immuno- gold TEM of 2A10: R21 complexed in different ratios and un-complexed R21 as a control. Arrows indicate staining of mAb 2A10 complexed with R21 VLP. Scale 100-200nm.

To investigate the potential for R21 to form immune complexes (ICs) with the anti-NANP monoclonal antibody (mAb) 2A10, we incubated these components at various ratios (1:1, 1:2, 2:1, and 5:1) and analyzed IC formation using western blotting and immunogold labeling followed by electron microscopy. Western blot analysis confirmed that ICs were formed at all tested ratios. In blot (A), staining with an anti-HBsAg antibody (**Figure 2A**) verified the presence of the HBsAg component of the R21 fusion protein. In blot (b), staining with a secondary antibody against mouse IgG (**Figure 2B**) confirmed the presence of mAb 2A10. Both blots revealed a high molecular weight IC band (~190 kDa) across all ratios, indicating successful complex formation. When samples were treated with a reducing agent, a band of the expected size (~50 kDa) corresponding to the R21 monomer was observed in blot (a), confirming the integrity of R21. The presence of a ~190 kDa band in both untreated blots further validated the formation of high molecular weight ICs. Additional bands at lower molecular weights suggest the presence of smaller complexes.

Immunogold labeling results were consistent with these findings. Positive labeling was observed for ICs at ratios of 1:1, 1:2, and 2:1, whereas the 5:1 ratio exhibited some aggregation, and labeling was less distinct (**Figure 2C**). Unconjugated R21 appeared negative for labeling, further confirming the specificity of IC formation. For subsequent vaccination experiments, we selected a 1:1 ratio (R21: mAb 2A10), ensuring consistency with the standard vaccine dose of 1 µg R21 used in combination with the Matrix-M adjuvant. This ratio was deemed optimal for generating ICs and was used throughout the study to compare with the R21 + MM control group.

### 3.2 R21/MM administered via the SC route is immunogenic and more efficacious than the IM route

**Figure 3:**
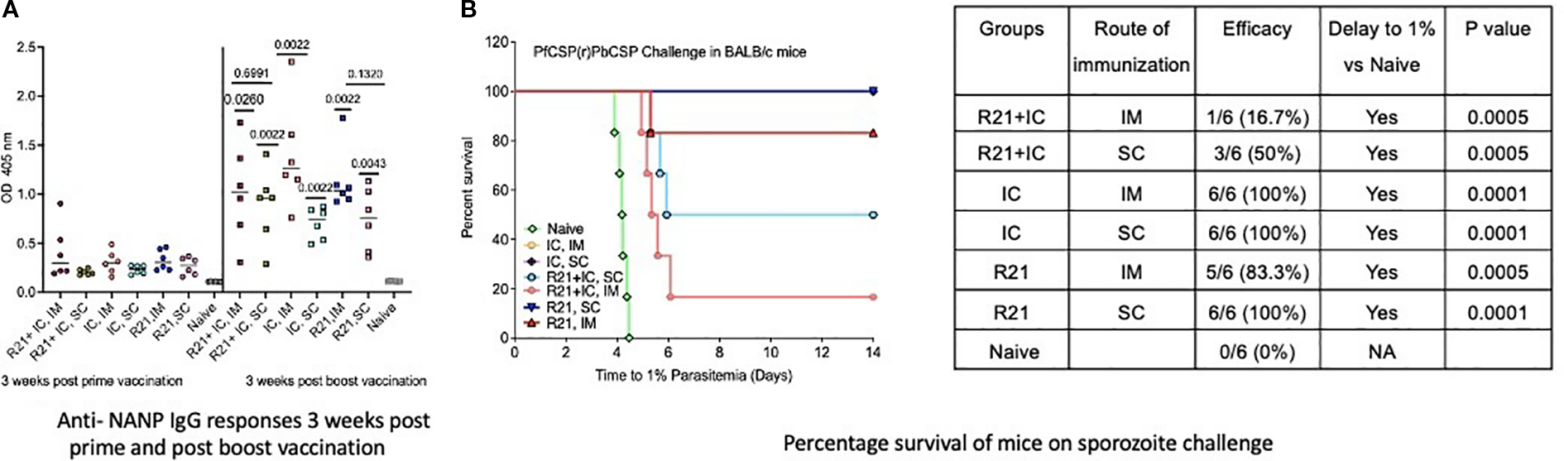
Immunogenicity and efficacy assessment of un-complexed R21 (R21+ MM), immune complexed R21 (mAb 2A10: R21 VLP + MM) and IC co-administered R21 (R21 + mAb 2A10: R21 VLP + MM) in BALB/c mice (n=6): **(A)** Anti-NANP IgG responses 3 weeks post prime and post boost vaccination as OD 405 values. Statistical significance shown above each group in black post boost is in comparison to anti-NANP IgG responses post prime. Between IC groups, IM vaccination induced significantly higher (** shown in orange) than SC vaccination. No significant difference was seen between the groups at same time point. **(B)** Percentage survival of mice when challenged with 1000 sporozoites (spz) intra-venously, 3 weeks post boost vaccination. Un-complexed R21 (+MM) show highest protection to spz challenge with SC group (100%) followed by IM group (83.3%). Both the groups immunized with immune- complexed R21 ( +MM) showed 100% protection on challenge. R21 co- administered with Immune- complexed R21 (+ MM) show a moderate protection of 50% via SC route and low protection (16.7%) via IM route. Analysis performed using Log-rank (Mantel-Cox) Test.

**Figure 4:**
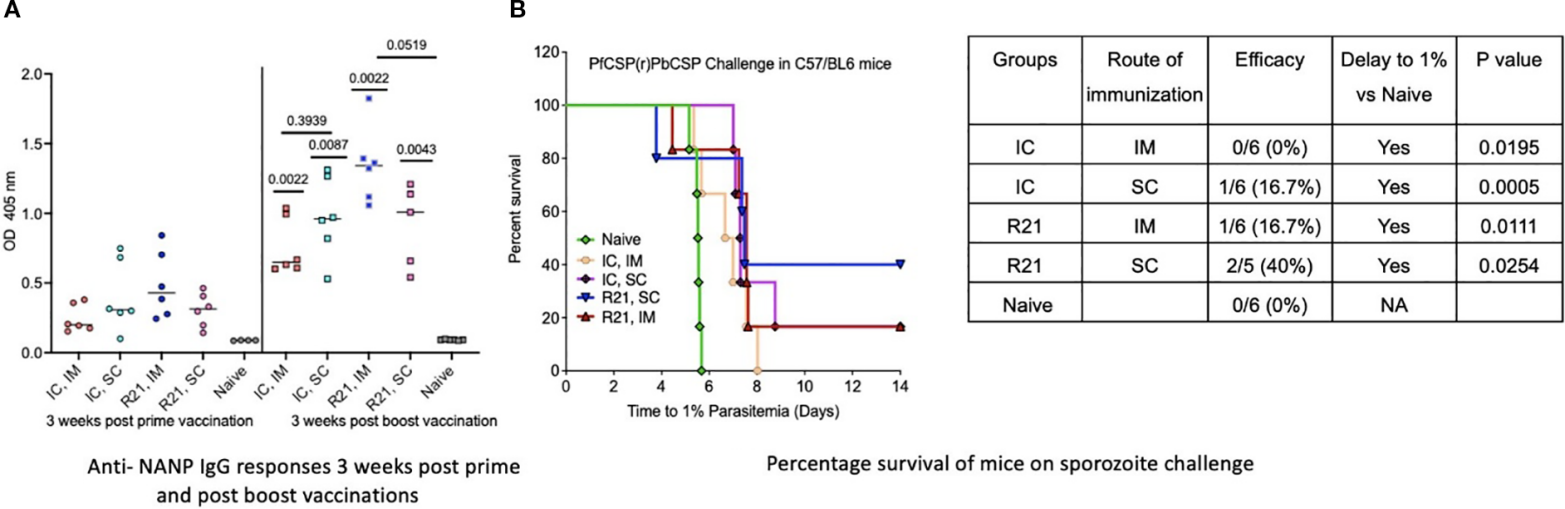
Immunogenicity and efficacy of un-complexed (R21 + MM), immune complexed (mAb 2A10: R21 VLP + MM) and VLP and IC co- administration (R21 + mAb 2A10: R21 VLP + MM) in C57/BL6 mice (n=6): **(A)** Anti-NANP IgG responses 3 weeks post prime and post boost vaccinations as OD 405 values. Statistical significance shown above each group are p values post boost is in comparison to anti-NANP IgG responses post prime. **(B)** Percentage survival when challenged with 1000 spz intra-venously, 3 weeks post boost vaccination. Mice immunized with un-complexed R21 (+MM) were protected on spz challenge, SC route (40%) and IM route (16.7%). Immune- complexed R21 via SC route conferred a low 16.7% protection, while via IM route failed to show any protection. Analysis performed using Log-rank (Mantel-Cox) Test.

Previously published data indicate that R21/MM is immunogenic and provides sterile protection against sporozoite challenge in BALB/c mice with three immunizations (18). In this study, we investigated the immunogenicity and protective efficacy of R21/MM administered via the subcutaneous (SC) route compared to the intramuscular (IM) route, using a two-dose vaccination regimen. Experiments were conducted in both BALB/c and C57BL/6 mouse strains, the latter strain being more difficult to protect in sporozoite challenge models (17). Both mouse strains exhibited significantly higher anti-NANP antibody responses following the boost vaccination (p=0.0043 for BALB/c and C57BL/6 mice) compared to after the first dose, regardless of the route of administration. However, no significant differences in anti-NANP levels were observed between SC and IM groups three weeks post-boost (p=0.1320 for BABL/c mice and p=0.0519 for C57/BL6 mice) (**Figures 3A**, **4A**). In challenge experiments, SC vaccination conferred superior protection and delayed parasitemia compared to IM vaccination. In BALB/c mice, SC vaccination achieved 100% protection, while IM vaccination provided 83.3% protection. Additionally, SC vaccination in C57BL/6 mice resulted in higher protection (40%) compared to the IM group (16.6%) (**Figures 3B**, **4B**). A significant delay to 1% parasitemia was observed in the SC-vaccinated (p=0.0001) and IM- vaccinated group (p=0.005) in comparison to the naive group. For C57BL/6 mice, the delay was significant compared to the naïve group (p=0.0254 for SC and p=0.0111 for IM group), but the difference between SC and IM groups remained non-significant (**Figures 3B**, **4B**). These results demonstrate that R21/MM administered via the SC route is not only immunogenic but also provides some enhanced protection against sporozoite challenge compared to the IM route, particularly in BALB/c mice.

### 3.3 R21:2A10 IC/MM is immunogenic and induces sterile protection in BALB/c mice

To evaluate the potential enhancement of anti-NANP antibody levels, BALB/c mice were vaccinated twice at a three-week interval with the R21:2A10 immune complex (IC) adjuvanted with Matrix-M (MM). Vaccination was administered via either the intramuscular (IM) or subcutaneous (SC) route. Both IM (p=0.0022) and SC groups (p=0.0022) demonstrated significantly higher anti-NANP IgG levels three weeks after the booster dose, with no significant differences observed between the two routes of administration (**Figure 3A**). Upon challenge with sporozoites, sterile protection was achieved in both IC-vaccinated groups, regardless of the route of immunization (**Figure 3B**). Additionally, a significant delay to 1% parasitemia (p=0.0001) was observed in IC-vaccinated mice compared to the naïve group. These results demonstrate that the R21:2A10 IC/MM formulation is highly immunogenic and capable of inducing sterile protection in BALB/c mice, irrespective of the route of administration.

### 3.4 Co-administration of R21/MM with IC/MM is immunogenic but less protective than IC/MM alone in BALB/c mice

Previous studies, such as those by Diamos et al. (13) using a Zika virus model, have demonstrated that co-delivery of virus-like particles (VLPs) with immune complexes (ICs) significantly enhances antibody responses and *in vitro* virus neutralization. Here, we investigated whether this co-administration strategy could be applied to malaria, a parasitic disease. BALB/c mice were vaccinated twice, at three-week intervals, with 1 µg R21 co-delivered with IC (R21: mAb 2A10) and adjuvanted with Matrix-M (MM). Both the intramuscular (IM) and subcutaneous (SC) groups showed a significant increase in anti-NANP antibody levels three weeks after the booster dose (p=0.0260 for IM and p=0.0022 for SC group), compared to levels three weeks after the prime dose. However, there were no significant differences in antibody levels between the two groups post-boost (p=0.6991) (**Figure 3A**).

Upon challenge, partial protection was observed: 16.7% in the IM group and 50% in the SC group (**Figure 3B**). Both groups exhibited a significant delay to 1% parasitemia (p=0.0005) compared to the naïve group. Despite significantly higher anti-NANP titers post-boost, with levels comparable to those seen in the R21: IC (IM)/MM group, the efficacy of co-administration was markedly lower. These findings suggest that while co-administration of R21/MM with IC is highly immunogenic, it does not achieve the level of protection conferred by IC/MM alone. Based on these results, we further investigated the immunogenicity and efficacy of the two IC/MM groups (via IM and SC routes) that provided sterile protection in BALB/c mice, extending the study to C57BL/6 mice to assess their potential in a more challenging model.

### 3.5 R21:2A10 IC/MM is immunogenic but partially protective in C57BL/6 mice when administered subcutaneously

To evaluate the immune response and efficacy of R21:2A10 immune complex (IC)/MM in a different mouse strain, we selected the two vaccination groups that conferred sterile protection in BALB/c mice and applied the same vaccination and challenge regimen in C57BL/6 mice. This strain is consistently more difficult to protect in malaria challenge studies. Both IC groups (IM and SC) elicited significantly higher anti-NANP antibody responses three weeks after the booster vaccination (p=0.0022 for IM and p=0.0087 for SC group) compared to levels observed three weeks post-prime (**Figure 4A**). No significant difference in antibody levels was observed between the IM and SC groups post-boost (p=0.3939). Following challenge, partial protection was observed in the SC group (16.7%), whereas the IM group did not confer measurable protection (**Figure 4B**). However, both groups exhibited a significant delay in reaching 1% parasitemia compared to the naïve control group (p=0.0195 for IM and p=0.0005 for SC route group). These findings demonstrate that while R21:2A10 IC/MM is highly immunogenic in C57BL/6 mice, its protective efficacy is limited, with partial protection observed only in the SC group. This highlights the need for further investigation to enhance efficacy in this challenging model.

## 4 Discussion

Achieving improved immune responses and reducing the number of required vaccinations remains a goal for many vaccines. The R21 vaccine has demonstrated high-level sterile protection with three intramuscular (IM) immunizations in preclinical studies. In human trials, three doses of R21/MM have shown unprecedented efficacy for a malaria vaccine, achieving 75% protection in African children. A fourth dose a year later is used to maintain protective anti-NANP antibody levels and high efficacy. To address this, we explored whether immunogenicity and efficacy could be maintained or enhanced with reduced number of immunizations (3 immunizations, 10μg twice followed by 2μg) which provided about 77% sterile efficacy in human challenge studies (19) and recent field trials in children (Datoo et al. unpublished data). In this pre-clinical study, with the aim to reduce number of doses, we administered R21/MM via the subcutaneous (SC) route, either un-complexed, as an immune complex (IC) with monoclonal antibody 2A10 or co-administered with a pre-formed R21: mAb 2A10 immune complex (R21/MM + IC (R21:2A10)).

The subcutaneous route of R21/MM administration demonstrated superior short- term protective efficacy in both BALB/c (100%) and C57/BL6 (40%) only with two doses. Despite deferring efficacies, there was no significant difference between anti-NANP IgG responses post boost both mice strains (20). This can be possibly attributed to fact that in BALB/c mice the immune response is Th2 biased contributing to B cell proliferation, differentiation and affinity maturation of antibodies, in comparison to Th1 biased in C57BL/6 mice (21, 22).

The IC platform has been successfully employed in poultry vaccines and has shown promise in preclinical and clinical studies for human diseases. This study aimed to generate an R21 VLP:2A10 mAb immune complex targeting the immunodominant NANP repeats and compare the immune responses elicited via different routes of administration against the control vaccine R21 adjuvanted with MM via the IM route. The optimal R21:2A10 ratio was determined through western blot analysis and immunogold TEM, confirming successful IC formation. Based on previous preclinical studies using a standard dose of 1 µg R21, we selected a 1:1 ratio (1 µg each of R21 and 2A10) for immunization experiments.

The primary aim of the VLP + IC co-administration study was to assess potential enhancements in antibody levels and short-term protective efficacy. Despite promising *in vitro* results from previous Zika and HPV studies, this approach failed to improve immunogenicity or efficacy in malaria. Notably, IC/MM groups administered via both IM and SC routes, as well as the R21/MM -only group via SC, achieved 100% protection.

These findings indicate that while VLP + IC co-administration generates high immunogenicity, it possibly does not achieve the balance of avidity and protective antibody isotype levels observed with IC/MM alone. Functional assays evaluating these aspects will be needed to understand these results. Importantly, this study represents the first report of R21/MM administered via the SC route in a challenge experiment compared to the standard IM route. The observed short- term protection with two SC immunizations highlights the potential value of investigating the underlying mechanisms and durability of antibody responses over extended periods. With a number of subcutaneously delivered licensed vaccines like DEN4CYD (Dengvaxia) and PPSV23 (Pneumovax) (23) that have shown durable protective immune responses, delivering R21/MM via SC route to achieve high efficacy with reduced doses holds a translational potential to humans. A key limitation of this work is the absence of a durability assessment. We did not evaluate responses beyond the immediate post-immunization period; therefore, maintenance of protection and antibody quality over time, as well as potential dose-sparing across longer intervals, remain unknown.

Future directions include exploring more stable methods of IC generation, such as fusion proteins where the antibody is directly linked to the VLP. This approach has been successfully implemented in plant-based systems (13) and could potentially be adapted for the existing R21 VLP platform produced using *Pichia pastoris*. The design of IC as a fusion protein can be altered to express only the Fc region of the antibody along with the antigen. This could minimize the possibility of formation of secondary structures that might affect the Fc function.

In summary, we identify here possible future modifications of the current mode of formulation and delivery of the high efficacy R21/MM vaccine that are of interest for potential new deployment strategies.

## Data Availability

The original contributions presented in the study are included in the article/supplementary material. Further inquiries can be directed to the corresponding author.
